# Molecular Interactions Between Reactive Oxygen Species and Autophagy in Kidney Disease

**DOI:** 10.3390/ijms20153791

**Published:** 2019-08-03

**Authors:** Gur P. Kaushal, Kiran Chandrashekar, Luis A. Juncos

**Affiliations:** 1 4301 West Markham, Slot 501, Little Rock, AR 72205, USA; 2 Renal Section, Central Arkansas Veterans Healthcare System Little Rock, Arkansas and Division of Nephrology, Department of Internal Medicine, University of Arkansas for Medical Sciences, Little Rock, AR 72205, USA

**Keywords:** reactive oxygen species, oxidants, autophagy, acute kidney injury, chronic kidney disease, diabetic nephropathy, mTORC1, AMPK

## Abstract

Reactive oxygen species (ROS) are highly reactive signaling molecules that maintain redox homeostasis in mammalian cells. Dysregulation of redox homeostasis under pathological conditions results in excessive generation of ROS, culminating in oxidative stress and the associated oxidative damage of cellular components. ROS and oxidative stress play a vital role in the pathogenesis of acute kidney injury and chronic kidney disease, and it is well documented that increased oxidative stress in patients enhances the progression of renal diseases. Oxidative stress activates autophagy, which facilitates cellular adaptation and diminishes oxidative damage by degrading and recycling intracellular oxidized and damaged macromolecules and dysfunctional organelles. In this review, we report the current understanding of the molecular regulation of autophagy in response to oxidative stress in general and in the pathogenesis of kidney diseases. We summarize how the molecular interactions between ROS and autophagy involve ROS-mediated activation of autophagy and autophagy-mediated reduction of oxidative stress. In particular, we describe how ROS impact various signaling pathways of autophagy, including mTORC1-ULK1, AMPK-mTORC1-ULK1, and Keap1-Nrf2-p62, as well as selective autophagy including mitophagy and pexophagy. Precise elucidation of the molecular mechanisms of interactions between ROS and autophagy in the pathogenesis of renal diseases may identify novel targets for development of drugs for preventing renal injury.

## 1. Introduction

Reactive oxygen species (ROS) are short-lived oxygen-containing molecules that are generated by enzymatic and non-enzymatic redox reactions during cellular aerobic metabolism [1,2]. ROS are comprised of oxygen free radicals and non-radical derivatives of oxygen, including superoxide anion (O_2_•−), hydroxyl (OH•), and hydrogen peroxide (H_2_O_2_). Oxygen free radicals are formed by partial reduction of molecular oxygen, generating oxygen with one or more unpaired electrons, or by complete reduction of oxygen, by accepting four electrons, resulting in the formation of water. ROS were originally considered deleterious and thought to function exclusively as molecules damaging to cellular components. However, during the past two decades or so, many studies have documented that ROS are not just unwanted byproducts of cell metabolism, but play a pivotal role in both physiological processes and the pathogenesis of diseases, including renal injury. Under normal physiological conditions, basal levels of ROS maintained by redox homeostasis regulate signal transduction pathways involved in numerous biological cellular responses, including cell proliferation, growth factor signaling, immune responses, differentiation, and autophagy [3,4]. To maintain cellular redox homeostasis, mammalian cells produce antioxidant enzymes and non-enzyme agents including superoxide dismutase (SOD), catalase, peroxidases, glutathione, and thioredoxin, which antagonize and detoxify ROS. SOD is a key antioxidant enzyme system that converts O_2_•− to H_2_O_2_ and oxygen; subsequently, catalase or the glutathione systems reduce H_2_O_2_ to H_2_O. All of the three isoforms of SOD are expressed in the kidney [5]. Antioxidant enzymes, including SOD, are decreased in animal models of acute kidney injury (AKI) [6,7,8] and dysregulate redox homeostasis. Dysregulation of ROS or elevation of ROS due to increased ROS/reactive nitrogen species (RNS) generation and/or decreased local antioxidant defenses results in oxidative stress. Thus, when oxidative stress exceeds the capacity of the cell to scavenge ROS, oxidative damage of cellular components, including lipids, proteins, nucleic acids, and carbohydrates, occurs [4,9].

One of the most important biological responses in the cell that is regulated by ROS and oxidative stress is autophagy. Autophagy is an evolutionarily conserved, dynamic process of degradation of intracellular organelles and macromolecules by lysosomal hydrolases. The degraded components, including free fatty acids and amino acids, are recycled to synthesize new proteins, organelles, and energy required by the cell. Therefore, autophagy is involved in the clearance and recycling of damaged organelles, as well as misfolded and aggregated proteins, to preserve cellular homeostasis [10,11]. A wide variety of internal and external cellular stresses, including oxidative stress, activate autophagy as an adaptive response to combat stress and prevent stress conditions. Stress-induced autophagy generally provides a protective role by eliminating and recycling damaged macromolecules, protein aggregates, and dysfunctional organelles [12,13,14]. Organelle-specific autophagy, known as selective autophagy, eliminates and recycles damaged and dysfunctional organelles, including mitochondria, peroxisomes, lysosomes, endoplasmic reticulum (ER), and even the nucleus [15]. Although oxidative stress-induced autophagy is considered to be primarily protective, the ultimate protective effect may depend on the relative activation and completion of the autophagy process. Ideally, optimal autophagic flux should ensure the autophagy-mediated supply of bioenergetic molecules (e.g., ATP) and break down products (e.g., amino acids and fatty acids) to maintain cellular biosynthesis. However, impaired autophagic flux or prolonged activation of autophagy may result in cell death due to disruption of cellular homeostasis [16,17]. The pro-survival function may also depend on the autophagic suppression of the stress-mediated cell death pathways, including apoptosis and regulated necrosis [16,17,18]. In addition, the ROS produced upon degradation of ferritin by autophagy promote the cell death process known as ferroptosis [19].

The pathogenesis of kidney diseases is associated with oxidative stress as well as autophagy induction. The role of autophagy in acute kidney injury and progressive renal diseases has been reviewed recently [20,21,22]. This article summarizes the current understanding of the molecular mechanisms by which ROS regulate autophagy in kidney disease.

## 2. Generation of Reactive Oxygen Species (ROS)

**Figure 1:**
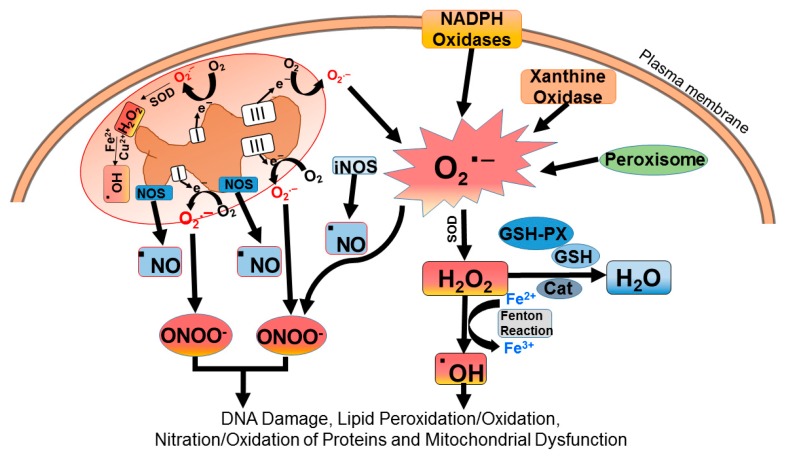
Generation of reactive oxygen species (ROS) in the cell. ROS are generated by enzymatic and non-enzymatic redox reactions during cellular metabolism under normal and pathological conditions. Mitochondria, plasma membrane, peroxisomes, and cytosol first generate the superoxide anion (O_2_•−), which becomes the precursor free radical for the generation of other ROS molecules. Cytosolic CuZN superoxide dismutase (SOD) and mitochondrial MnSOD, which are expressed in the kidney, dismutate O_2_•− to H_2_O_2_, which yields highly reactive hydroxyl radicals (OH•) by interaction with reduced transition metal ions (such as Fe and Cu) in a Fenton reaction. In addition to ROS, cells also generate reactive nitrogen species (RNS). The major RNS include nitric oxide (•NO), peroxynitrite (ONOO^−^), and nitrogen dioxide (•NO_2_). Nitric oxide (•NO) is produced by three isoforms of nitric oxide synthase (NOS), all of which are expressed in the kidney. ROS produced cause oxidative damage, including DNA damage, lipid and protein oxidation, protein nitration, and mitochondrial dysfunction.

In the cell, superoxide anion (O_2_•−) is the first free radical generated by mitochondria, plasma membrane, peroxisomes, and cytosol [9,23] (Figure 1).

The mitochondria are one of the primary sources of O_2_•− in living cells. In the mitochondrial respiratory electron transport chain, complex I and complex III were initially identified as sites involved in O_2_•− generation due to “electron leakage” to molecular oxygen [1,23]. Recently, additional distinct sites have been identified that may generate O_2_•− in mammalian mitochondria [24]. In the plasma membrane and neutrophils, O_2_•− is formed by the transfer of electrons to molecular oxygen through an enzymatic reaction catalyzed by the membrane-bound NOX family of NADPH oxidases [25,26,27]. The cytosolic enzyme xanthine oxidase [28] is a well-studied ROS-producing enzyme that catalyzes xanthine and hypoxanthine oxidation to uric acid and transfers monovalent electron to molecular oxygen to produce O_2_•− [28,29]. Once O_2_•− is generated, it becomes a precursor for the formation of other ROS in the cell (Figure 1). SOD converts O_2_•− to H_2_O_2_, which can generate highly reactive OH• by interacting with transition metal ions (such as Fe and Cu), and extremely reactive hypochlorous acid, catalyzed by myeloperoxidase [30]. In addition to ROS, cells also generate RNS. The major RNS include nitric oxide radicals (•NO), peroxynitrite (ONOO^−^), and nitrogen dioxide (•NO_2_). The •NO radical is produced by three isoforms of nitric oxide synthase (NOS), all of which are expressed in the kidney [31]. It serves a role as a potent vasodilator and is involved in the regulation of hypertension. Increasing •NO levels can further react with O_2_•− to produce ONOO^−^, which can cause protein nitration [32].

## 3. Role of ROS in the Pathogenesis of Kidney Disease

Many studies have documented the important roles of ROS in the pathogenesis of AKI and chronic kidney disease (CKD), in animal models as well as in patients. The kidney is rich in mitochondria, with a high respiration rate to meet metabolic demand, and, therefore, it is highly vulnerable to generation of ROS and oxidative damage [33,34]. The increased production of ROS causes inflammation and tissue damage in ischemia-reperfusion (IR) [35,36,37,38] and toxic AKI [39,40,41,42], including mitochondrial ROS generation in renal IR [38,43] and cisplatin nephrotoxicity [44,45]. In renal IR, H_2_O_2_ production is increased four-fold [46]. ROS also contribute to the progression of kidney fibrosis [47,48] and the progression of CKD [49]. In diabetic nephropathy (i.e., hyperglycemic conditions in a diabetic milieu), ROS originate primarily from mitochondria and NADPH oxidase [50], but other sources, including glucose autooxidation, advanced glycation end products, activation of the polyol (sorbitol) pathway, and activation of the protein kinase C signaling pathway, also contribute to the production of ROS [50,51]. In cultured human podocytes, nicotine increased ROS generation and caused podocyte apoptosis, as well as associated downstream MAPK signaling [52]. In patients, increased oxidative stress has been reported in the early stages of CKD [53,54], and there is a positive correlation between oxidative stress and kidney disease progression [55,56]. Moreover, ROS generated by NOX and xanthine oxidase contribute to oxidative stress in CKD patients. For example, higher activities of NOX [57,58] and xanthine oxidase [59,60] have been reported in CKD and dialysis patients. Furthermore, in patients with advanced stages of CKD, oxidative stress is linked with complications including hypertension, inflammation, and atherosclerosis [49]. Thus, in pathogenesis of renal diseases, ROS may play vital roles in modulating signal transduction pathways involved in numerous biological cellular responses, including autophagy.

## 4. ROS-Mediated Regulation of Autophagy and Its Impact in Kidney Disease

### 4.1. Impact of Oxidants and Antioxidants on Autophagy in Kidney Disease

Many compounds that act as antioxidants have an impact on autophagy in kidney disease (Table 1). Rutin, an antioxidant flavonol glycoside, attenuated gentamicin-induced nephrotoxicity by reducing oxidative stress, autophagy, and inflammation in rats [61]. Rutin also protected against IR injury in rats [62]; however, whether the protection was mediated by autophagy is not known. N-acetylcysteine ameliorated gentamicin-induced nephrotoxicity by reducing ROS and enhancing autophagy [63]. Ferulic acid, a naturally occurring phenolic compound that possesses antioxidant activity, provided a beneficial effect in streptozotocin-induced diabetic nephropathy in rats by inhibiting ROS generation, nuclear factor (NF)-κB activation, stress signaling pathway (p38, c-Jun N-terminal kinase (JNK), and extracellular signal-regulated kinase 1/2) activation, and by promoting autophagy [64]. Cadmium-induced oxidative stress and subsequent cytotoxicity in the proximal tubule was prevented by trehalose by restoring autophagic flux [65]. Berberine, a quaternary ammonium salt from the protoberberine group of isoquinoline alkaloids that possesses potent antibiotic, anti-inflammatory, and antioxidant activity, ameliorated cisplatin-induced renal function and histopathological changes and suppressed oxidative stress, inflammation, autophagy, and apoptosis [66]. Celastrol, a bioactive triterpenoid that has antioxidant, anti-inflammatory, and anti-apoptotic properties [67,68], induced heme oxygenase (HO-1)-mediated autophagy and blocked high glucose-induced injury, inflammation, and insulin resistance in podocytes [69]. In podocytes, HO-1-induced autophagy has been shown previously to provide a protective mechanism against high glucose-induced apoptosis [70]. HO-1, an endogenous antioxidant, reduced defective autophagy in cisplatin-induced AKI and protected against cisplatin-induced apoptosis in proximal tubules [71]. Another study showed that HO-1 reduced H_2_O_2_-induced excessive autophagy and protects glomerular mesangial cells [72].

ROS-induced autophagy provided a protective role in aldosterone-induced podocyte injury [73], and targeting autophagy in podocytes may be a therapeutic strategy for podocyte injury. In addition, activation of ER stress by aldosterone-mediated ROS generation increased autophagy, which protected podocytes from apoptosis [74]. Chronic oxidative stress due to knockdown of manganese SOD induced autophagy and mitochondrial biogenesis following renal IR, thus protecting the kidneys against acute oxidative stress [75]. Additionally, ischemic postconditioning (POC), which reduces overproduction of ROS generation in renal tubular cells, provided a renoprotective effect against AKI and renal fibrosis through the activation of autophagy [76].

Induction of autophagy can also modulate ROS production in renal injury. For example, cisplatin treatment in autophagy-deficient renal proximal tubular cells increased ROS production, oxidative stress, and DNA damage [77], suggesting that autophagy provides protection by reducing ROS production and eliminating toxic oxidized protein aggregates and other macromolecules. Hyperoxaluria-induced renal tubular oxidative and calcium-oxalate-crystal-induced renal tubular epithelial cell injury was attenuated by inhibition of autophagy [78,79]. Another study provided evidence that ER stress-triggered autophagy protects against oxidative injury and ameliorates renal IR injury [80]. Correlative evidence on the role of ROS in autophagy in renal diseases has been previously reported [81], but, in these studies, molecular mechanisms of interaction between ROS and autophagy were not investigated. As described below, many studies have been now performed which provide evidence that ROS impact on autophagy is mediated by specific signaling pathways in renal diseases.

### 4.2. ROS-Mediated Oxidation of Autophagy-Related Proteins

**Figure 2:**
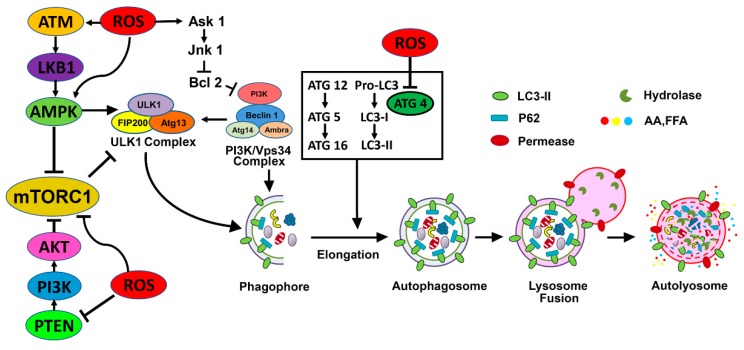
Regulation of autophagy by ROS. (**Top left**) Under conditions of oxidative stress, ROS oxidize cysteines located in the α and β subunits of AMPK, by activating AMPK and its downstream signaling. Members of the Bcl-2 family suppress autophagy by binding beclin-1 and preventing beclin-1 from forming the PI3 kinase/Vps34 complex of the autophagy pathway. ROS-mediated oxidation of Ask1 results in dissociation of the Bcl-2–beclin-1 interaction, making free beclin-1 available for the formation of PI3 kinase/Vps34 complex and subsequent autophagy activation. (**Top right**) H_2_O_2_ oxidizes the cysteine 81 residue of Atg4, which inactivates the protease to facilitate Atg8 in the formation of autophagosome. (**Bottom)** Oxidative stress also oxidizes mTORC1 and PTEN, which inhibits their activity and promotes autophagy. Suppression of mTORC1 and induction of AMPK promote ULK1 complex (ULK1, Atg13, FIP200, and Atg101) activation at the pre-autophagosomal assembly site (a certain domain of the ER) and initiates the autophagy process.

Although the specific interactions between autophagy and oxidative stress have not been elucidated fully, it is known that autophagy can be modulated by oxidative stress, generally enhancing its induction [82,83], and that antioxidants may prevent ROS-induced autophagy induction [84]. ROS-induced autophagy may lead to a different outcome of cell fate that may result in cell survival or cell death, depending on the severity of ROS exposure. Redox signaling, ROS-mediated oxidized macromolecules and organelles, and mild oxidative stress activate the autophagy pathway to eliminate damaged macromolecules, and protein aggregates to provide cytoprotection and maintain cellular homeostasis. Under starvation conditions, ROS originating from mitochondria and NADPH oxidases activate autophagy to provide protection from nutrient starvation by autophagic elimination of damaged mitochondria and other organelles [83,85]. In addition, increased generation of ROS can oxidize key cysteine residues in autophagy proteins and modify their function. A direct target of ROS in the autophagy pathway is cysteine protease Atg4, which is involved in the processing of ATG8. Oxidation of Atg4 by H_2_O_2_ at the critical Cys 81 residue inactivates the protease, thus facilitating autophagosome formation by Atg8 [86] (Figure 2). Furthermore, SQSTM1/p62 is an autophagic multidomain receptor containing redox-sensitive cysteine residues (Cys 105 and Cys 113), which promotes oligomerization of p62, which then assembles aggregates of ubiquitinated proteins and is recruited to the autophagosome by LC3 binding through the LC3-interacting region of p62 [87]. Oxidation of redox-sensitive cysteine residues in p62, therefore, can affect p62 function, which is required for selective autophagy.

### 4.3. ROS Impacts on the mTORC1-ULK1 and AMPK-mTORC1-ULK1 Signaling Pathways of Autophagy and Their Role in Kidney Disease

#### 4.3.1. ROS Impact on the mTORC1-ULK1 Pathway and Its Role in Kidney Disease

The interaction of the serine–threonine kinase mTORC1 with autophagy is mediated by the mTOR-ULK½ signaling pathway [88,89,90] (Figure 2). mTORC1 senses and responds to various forms of cellular stress, including oxidative stress, and is inactivated by most types of stress [91,92]. ROS influence mTORC1 activity [93,94], and thus may affect mTORC1 signaling and autophagy. mTORC1 is considered a negative regulator of autophagy because suppression of mTORC1 activates autophagy.

Inactivation of mTORC1 (e.g., nutrient starvation) activates the ULK1 complex of the autophagy pathway by dephosphorylation of ULK1 at Ser 757, which then permits ULK1 to phosphorylate its associated components Atg13 and FIP 200, resulting in ULK1 activation. Active ULK1 then activates downstream targets of the autophagy pathway, including members of VPS34 complex 1, promoting the synthesis of phosphatidylinositol 3-phosphate (PI3P) [95,96], a key player in membrane dynamics involved with the autophagosome preassembly machinery. In contrast, activation of mTORC1 in response to growth factors, amino acid load, nutrients, and increased energy levels promotes anabolic processes (e.g., protein synthesis, lipogenesis, cell growth, metabolism, and proliferation) and suppresses catabolic processes, including autophagy [91,97,98]. mTORC1 represses autophagy upon inhibiting the kinase activity of ULK½ by phosphorylation of ULK1 (Ser 638/758) and associated partner Atg13 (Ser 258) [99]. Thus, mTORC1 levels play a pivotal role in the induction and regulation of autophagy. Thioredoxin interacting protein (TXNIP), which induces ROS production, regulated tubular autophagy and mitophagy in a rat model of diabetic nephropathy through the mTOR signaling pathway [100]. In human glomerular mesangial cells, Ang II-induced oxidative stress promoted autophagy via mTORC1 signaling, and caused premature senescence [101]. The senescence-promoting effect of autophagy activation was attributed to the induction of excessive ROS production.

#### 4.3.2. ROS Impact on the AMPK-mTORC1-ULK1 Pathway and Its Role in Kidney Disease

AMP-activated protein kinase (AMPK) is an energy- and nutrient-sensitive kinase that is activated in response to a high AMP/ATP ratio [102], and stimulates autophagy by suppressing mTORC1 [88] and by phosphorylating ULK1 at multiple sites (Figure 2). AMPK phosphorylates tuberous sclerosis complex 2 (TSC2), thereby increasing TSC1/2 activity, which ultimately inhibits mTORC1 [103,104]. Additionally, in response to energy deprivation, AMPK activates the ULK½ complex directly by phosphorylating ULK1 at Ser 317 and Ser 777, activating the autophagy pathway [105,106]. AMPK-mediated phosphorylation of other ULK1 sites is not energy- or nutrient-dependent, but regulates the localization of the Atg9A protein, which plays a key role in the organization of the preautophagosomal/phagophore assembly site [107]. Beclin-1 in the phosphoinositide 3- kinase catalytic subunit type 3 (PI3KC3) complex is also phosphorylated by AMPK, favoring autophagy activation [106]. Phosphorylated ULK1/2 activates the class III PI3 kinase complex (PI3KC3–C1/VPS34 complex) by phosphorylating beclin-1 (Ser 15 and other sites) [96], the catalytic subunit VPS34 (Ser 249) [105], and the PI3KC3-C1-associated protein AMBRA1 (autophagy and beclin-1 regulator 1) [108] thereby promoting autophagy. ULK1 also promotes phosphorylation of FUNDC1 (FUN14 domain containing 1) protein, which is involved in the process of mitophagy [109]. Thus, AMPK-mediated activation of ULK½ initiating complex plays a crucial, multifaceted role in autophagy regulation.

AMPK regulates cellular metabolism and is a key component of the cellular adaptive responses to renal ischemia. Recent studies have suggested that pretreatment with AMPK activators (5-aminoimidazol-4-carboxamide-1-*β*-d-ribofuranoside (AICAR) and metformin) can protect against renal IR injury [110]. Inhibition of AMPK with compound C before IR abolished AMPK activation, worsened kidney injury, and diminished the expression of uncoupled protein 2 (UCP2) and sirtuin (SIRT)-3, but not that of stanniocalcin-1. A high-fat diet (HFD) leads to lipid accumulation and decreases the levels of AMPK in the kidney, resulting in lipotoxicity. Pharmacological activation of AMPK by AICAR prevents HFD-induced tubular cell structure impairment and the associated inflammation and fibrosis [111]. Additionally, fenofibrate-mediated upregulation of AMPK has been shown to provide protection from kidney injury both due to HFD-induced lipotoxicity [112] and in *db/db* mice [113]. Although HFD-induced renal tubular injury has been associated with impaired autophagy [114,115], it is not known whether activation of AMPK alleviates HFD-induced dysregulation of autophagy. ROS can activate AMPK and influence autophagy via multiple mechanisms (Figure 2). H_2_O_2_ can directly activate AMP kinase by oxidizing cysteine residues [116] of the α and β subunits, as well as indirectly, by increasing AMP levels (decreasing the ATP/AMP ratio) [117,118] or through oxidation of ataxia–telangiectasia mutated (ATM) protein kinase [119]. ROS- and RNS-mediated activation of ATM transduces further downstream signaling via ATM-AMPK-TSC2-mediated suppression of mTORC1, thus impacting autophagy [120,121]. As a result, in an ATM-, LKB1-, and TSC-dependent fashion, mTORC1 is repressed. Additionally, starvation-induced autophagy involves ROS-mediated activation of AMP kinase [122]. Moreover, preactivation of AMPK has been shown to provide protection from renal injury in models of IR- [110,123] and cisplatin-induced [124] AKI, and diabetic nephropathy [125].

Many agents that upregulate AMPK by redox signaling provide protection from renal injury via the AMPK/mTOR-regulated autophagy pathway (Table 2). In renal IR, quercetin, a flavonoid antioxidant and a potent scavenger of ROS [126], has been reported to increase AMPK-phosphorylation, inhibit mTOR activation, activate autophagy, and provide protection from renal injury [127]. Another compound, pioglitazone, enhanced antioxidant capacity [128] and protected against renal IR through the AMPK-regulated autophagy pathway [129]. Additionally, ω3-polyunsaturated fatty acid involved in the regulation of mitochondrial anion carrier protein uncoupling protein 2 (UCP2), and p22phox expression have been shown to cause enhanced antioxidant effects and provide protection from renal IR injury through AMPK-mediated autophagy in Fat-1 mice [130]. Oxidative stress plays an important role in induction of autophagy via the AMPK/mTOR signaling pathway during the pathogenesis of cisplatin nephrotoxicity. Cisplatin-induced AKI involves oxidative stress [44,45], and, in one model, treatment with ginsenoside Rb3 increased glutathione (GSH) content and superoxide dismutase (SOD) activity, reduced oxidative stress, and decreased autophagy via the AMPK/mTOR signaling pathway [131]. Deletion of a cytoprotective antioxidant, NAD(P)H: quinone oxidoreductase 1 (NQO1), displays cisplatin-induced enhanced autophagy via the AMPK/mTOR signaling pathway [132]. Other studies have shown that neferine [133] and metformin [134] ameliorated cisplatin-induced nephrotoxicity by enhancing autophagy via the AMPK/mTOR signaling pathway. Taken together, these studies suggest that increased oxidative stress enhances autophagy through the AMPK/mTOR signaling pathway in cisplatin-induced AKI. Many nephrotoxic agents that generate ROS inhibit PI3k-Akt-mTORC1 signaling and activate autophagy. For example, nephrotoxicity by ROS-producing bismuth nanoparticles (BiNPs) activates autophagy through the AMPK/mTOR pathway [135], and blockage of BiNP-induced ROS suppresses autophagy, suggesting that ROS produced by BiNPs are involved in the induction of autophagy. Additionally, BiNP-induced AKI is exacerbated when autophagy is inhibited, suggesting a protective role of autophagy [135]. In a sepsis model of cecal ligation and puncture, SIRT3 protected against AKI via AMPK/mTOR-regulated autophagy [136].

In a diabetic nephropathy model, metformin an inducer of AMPK protected against cisplatin-induced tubular cell apoptosis and AKI via AMPK-regulated autophagy induction [134]. Berberine, an isoquinoline alkaloid (an antioxidant and anti-inflammatory compound), enhanced AMPK activation and autophagy and reduced high glucose-induced apoptosis of mouse podocytes [137]. Ginsenoside Rg1 promoted protection of mouse podocytes from aldosterone-induced injury by inhibiting ROS generation and reduced aldosterone-induced autophagy via the AMPK/mTOR pathway in NRK-52E cells [138]. Mangiferin, a polyphenol glucoside antioxidant [139], prevented diabetic nephropathy progression and protected podocyte function via autophagy through the AMPK-mTOR-ULK1 pathway in diabetic rat glomeruli [140]. Another antioxidant, the saponin astragaloside IV, provided protection from podocyte injury via AMPK-regulated autophagy induction and SERCA2-dependent ER stress reduction in streptozotocin-induced diabetic nephropathy [141]. Cinacalcet, a type II agonist of the calcium-sensing receptor (CaSR) that increases the expression of SOD1 and SOD2 antioxidant enzymes and decreases the levels of urinary 8-hydroxy-deoxyguanosin and isoprostane, has been shown to attenuate diabetic nephropathy in *db/db* mice by modulation of autophagy through the CaMKKβ-LKB1-AMPK pathway [142]. Meanwhile, in Fat-1 mice, accumulation of ω-3 polyunsaturated fatty acids prevented renal IR injury through AMPK-mediated autophagy [130].

Angiotensin II (ANG II) activates autophagy in podocytes through the generation of ROS, as the ROS-mediated proautophagic effect of ANG II was inhibited by treatment with antioxidants and ginsenoside Rg1 inhibited ANG II-induced podocyte autophagy via the AMPK/mTOR/PI3K pathway [143]. In a murine model of experimental chronic obstructive pulmonary disease (COPD), chronic cigarette smoke (CS) exposure caused marked kidney injury, fibrosis, oxidative stress, mitochondrial dysfunction, and increased autophagic flux. Kidney injury and fibrosis were ameliorated in mice heterozygous for beclin-1 exposed to CS [144], suggesting that autophagy is a therapeutic target in renal injury due to CS.

### 4.4. ROS Impact on Keap1/Nrf2 System-Mediated Autophagy and Role in Kidney Disease

**Figure 3:**
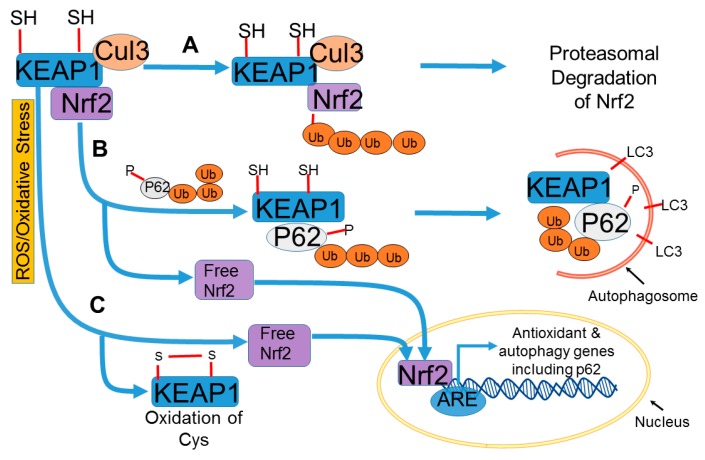
ROS regulation of Keap1/Nrf2/p62 signaling and its impact on autophagy. (**A**) Under normal basal conditions, Keap1 regulates Nrf2 and keeps its level low by ubiquitination and proteosomal degradation. (**B**) P62 has a binding site for Keap 1 and promotes autophagic degradation of Keap1, liberating Nrf2 and enabling Nrf2 to translocate to the nucleus to transactivate target genes to protect against oxidative stress and upregulate autophagy. (**C**) Under oxidative stress conditions, Keap1 is oxidized by ROS-mediated oxidation of cysteine thiols in the cysteine-rich Keap1-Cullin3-based ubiquitin ligase complex, which dissociates Nrf2 from Keap1 and enables Nrf2 to translocate to the nucleus to transactivate target genes, including antioxidant and autophagy genes.

Nuclear factor E2-related factor 2 (Nrf2) is a key transcription factor that regulates the expression of several genes encoding antioxidant and detoxifying enzymes to maintain cellular redox homeostasis and to provide cellular defense against oxidative stress [145,146] (Figure 3).

Nrf2 function in controlling the transcription of antioxidant genes is mediated through interaction with antioxidant-response element (ARE) [147]. Under normal conditions, Nrf2 levels in the cell are kept low due to proteasomal degradation upon binding of Nrf2 to Keap1 (Kelch-like erythroid cell-derived protein with CNC homology (ECH)-associated protein 1) bound with the adaptor Cullin3-based ubiquitin ligase, which ubiquitinates Nrf2 [146]. In response to oxidative stress, cysteine thiols in the cysteine-rich Keap1-Cullin3-based ubiquitin ligase complex are modified, which dissociates Nrf2 from Keap1, and free Nrf2 translocates to the nucleus to transactivate target genes [146,148]. Nrf2 itself possesses a redox-sensitive nuclear exporting signal in the transactivation domain that promotes translocation of Nrf2 to the nucleus in response to oxidative stress [149]. These studies suggest that in response to oxidative stress, Nrf2 can translocate to the nucleus independent of binding to Keap1. The selective autophagy substrate p62 interacts with the Nrf2-binding site on Keap1, and the phosphorylation of p62 markedly increases the binding affinity of p62 for Keap1. In this process, p62 is first assembled on the aggregates of ubiquitinated proteins and organelles, and subsequently phosphorylated in an mTORC1-dependent manner before binding to Keap1 with high affinity, allowing release of Nrf2, translocation to the nucleus, binding with ARE, and subsequent transcriptional activation of Nrf2 target genes [150]. The Keap1-p62 complex formed in this process is recruited to the autophagosome by LC3 via binding to LC3-interacting region of p62, and subsequently degraded by autophagy [151,152,153] (Figure 3). Several studies have provided evidence that the Keap1-Nrf2 pathway is intimately linked to autophagy through the autophagy adaptor and LC3-binding protein p62 [151,154,155,156,157]. Nrf2 is also able to upregulate many autophagy genes (*Atg3*, *Atg5*, *Atg7*, *SQSTM1,* and *LAMP2A)* by binding to the ARE sequence in the promoter region in response to oxidative stress [158,159]. Therefore, Nrf2 has been reported to activate both macroautophagy and chaperone-mediated autophagy. A recent study demonstrated that upon prolonged exposure of oxidative stress by *tert*-butyl hydroperoxide (TBHP) in HEK293T cells and *Caenorhabditis elegans*, Nrf2 negatively regulates autophagy through delayed down-regulation of AMPK [160]. Silencing of *NRF2* gene during prolonged exposure to TBHP results in the continuous up-regulation of AMPK. These studies suggest that Nrf2 has a direct impact on autophagy through AMPK, and that Nrf2 negatively regulates autophagy upon down-regulation of AMPK.

In AKI models, increased levels of Nrf2 protein and expression of several antioxidant genes are observed during IR and toxic AKI. Nrf2 provided a protective role in models of AKI and CKD, including diabetic nephropathy [145,161,162,163,164,165,166]. Lycopene, known to play an antioxidant role, ameliorated atrazine-induced nephrotoxicity by upregulating Nrf2, AMPK, and autophagic flux, as well as by decreasing oxidative stress and p62 levels [167]. Schisandrin B, isolated from the fruit of *Schisandra chinensis*, provided a protective role in cyclosporine (CsA)-induced nephrotocxicity, attenuated CsA-induced nephrotoxicity by activating Nrf2, preventing ROS accumulation and oxidative stress, and allowed recovery of CsA-induced blockade of autophagic flux [168]. Cyclic helix B peptide, a tissue-protective peptide mimicking the 3D structure of erythropoietin, which has been reported to ameliorate IR [169], protected HK-2 cells from H_2_O_2_-induced injury through activation of the Nrf2 signaling pathway and autophagy [170]. However, in this study, the link between Nrf2 and autophagy was not determined.

### 4.5. ROS Impact on Forkhead Box O (FOXO)-Mediated Autophagy and Its Role in Kidney Disease

Forkhead Box O (FOXO) transcription factors are also a target of oxidative stress. Oxidative stress indirectly activates FOXO by ROS-mediated activation of JNK kinase, which phosphorylates both FOXO and 14-3-3 protein, promoting the release of FOXO factors, leading to subsequent nuclear translocation and transcriptional activity [171]. In kidneys from a unilateral ureteral obstruction model (UUO) at day 7, FOXO3 activation increased both the mRNA and protein levels of key autophagy proteins including Ulk1, beclin-1, Atg9A, Atg4B, and Bnip3 [172], suggesting that FOXO3 is an important regulator of autophagy in renal tubular epithelial cells. In renal IR injury, hypoxia-mediated inhibition of prolyl hydroxylation prevents FOXO3 degradation resulting in FOXO3 accumulation and activation in tubular cells. Hypoxia-activated HIF-1α contributes to FOXO3 activation and functions to protect the kidneys, since tubular deletion of HIF-1α decreased FOXO3 activation and caused more severe tubular injury and interstitial fibrosis, suggesting that FOXO3 activation protects kidneys from IR injury [173].

### 4.6. ROS Impact on Selective Autophagy and Its Role in Kidney Disease

#### 4.6.1. ROS Impact on Mitophagy and Role in Kidney Disease

Selective degradation and elimination of damaged mitochondria occurs through the process of mitophagy to maintain quality control of mitochondria and cellular homeostasis [174]. Pink1/Parkin and Bnip3/Nix/Fundc1 are two known pathways for the selective elimination of damaged mitochondria. The Pink1/Parkin pathway is relatively well characterized, and is activated in response to depolarized and dysfunctional mitochondria. Pink1 is a serine/threonine kinase that is constitutively degraded by the stromal processing peptidase in the normal mitochondria, but it accumulates on the surface of damaged and depolarized mitochondria. Accumulated Pink1 then recruits E3 ubiquitin ligase Parkin and activates it by phosphorylation [175,176,177]. Pink1 can also phosphorylate ubiquitin, which promotes further activation of Parkin [175,178]. Active Parkin then ubiquitinates mitochondrial surface proteins, and the ubuiquitinated mitochondria are delivered to the autophagosome via p62 containing ubiquitin-binding and LIR structural domains, facilitating autophagic clearance of the damaged mitochondria [179,180].

Mitochondrial dysfunction and increased oxidative stress are associated in the pathogenesis of both chronic and acute kidney diseases [181]. Mitochondria are not only the primary source of ROS, but they also are targeted by ROS, which results in mitochondrial dysfunction and in turn produces more ROS and oxidative stress. Damaged and dysfunctional mitochondria may induce mitophagy, which, in turn, may decrease mitochondrial ROS generation. Recent studies have implicated ROS in promoting the translocation of Parkin to mitochondria for the induction of Parkin/PINK1-dependent mitophagy [182].

In models of AKI and CKD, Pink1/Parkin-mediated mitophagy induction has been reported to play a beneficial role and ROS have been shown to play an important role in regulating mitophagy in renal injury. In renal IR injury, *Pink1* and *Park2* single- and double-knockout mice had enhanced injury, mitochondrial damage, reactive oxygen species production, and inflammatory response compared to wild-type mice [183]. Another study reported that sestrin-2 and BNIP3 are upregulated in proximal tubular cells, and that the p53-sestrin-2 and HIF-1α-BNIP3 pathways are involved in the induction of mitophagy during IR injury in vivo [184]. In cisplatin nephrotoxicity, Pink1 or Park2 deficiency caused more apoptosis, mitochondrial dysfunction, and tissue damage both in vitro and in vivo [185,186]. However, a recent study showed that mitophagy induced by *Panax notoginseng saponins* (PNS) ameliorated cisplatin-induced nephrotoxicity via a HIF-1α/BNIP3/beclin-1 signaling pathway [187]. In contrast-induced AKI, Pink1/Parkin-mediated mitophagy was also induced in renal tubular epithelial cells in vitro [188] and in vivo [188,189,190] models. The protective role of mitophagy was demonstrated using *Pink1* or *Park2* knock out mice, and it was revealed that deficiency of Pink1/Parkin-mediated mitophagy elevated mitochondrial ROS production, DNA oxidative damage, and NLRP3 inflammasome activation [188]. ROS were generated in response to cadmium induced Pink1/Parkin-mediated mitophagy in mouse kidneys [191]. In renal tubular cells of diabetic kidney disease, increased mitochondrial ROS and mitochondrial fragmentation were associated with reduced Pink1/Parkin-mediated mitophagy and enhanced apoptosis. MitoQ, a mitochondria-targeted antioxidant, partially restored mitophagy and protected against tubular injury in *db/db* mice [192]. In diabetic nephropathy, *Pink1* transcriptional activity is controlled by the antioxidant FOXO 1, which promotes mitophagy and protects injured podocytes under HG conditions [193]. High myo-inositol oxygenase (MIOX) expression in the renal tubules of diabetic mice results in increased production of ROS, mitochondrial fragmentation, and impaired mitophagy. Supplementation of MIOX inhibitor d-glucarate decreased MIOX expression, attenuated tubular damage, reduced oxidative stress, restored mitophagy, and improved renal functions [194]. These studies suggest that inhibition of MIOX may play an important role in mitochondrial quality control and mitophagy in the pathogenesis of diabetic kidney disease (DKD), and that d-glucarate may serve as a potential therapeutic agent for the amelioration of DKD.

#### 4.6.2. ROS Impact on Pexophagy and Its Role in Kidney Disease

Selective degradation and elimination of damaged or dysfunctional peroxisomes by autophagy occurs through the process of pexophagy [195]. Peroxisome organelles in the cell have enzymatic systems to generate various ROS, including O_2_•−, H_2_O_2_, •NO, •OH, and ONOO^−^, as byproducts of their normal lipid metabolism, and to maintain redox balance by counter-balancing the production of peroxisomal antioxidant enzymes [196]. The process of pexophagy requires ubiquitination of peroxisomal biogenesis factor five (Pex5), an import receptor for peroxisomal matrix proteins [197], for subsequent binding to p62 and targeting to autophagosome for degradation. In this process, ROS-activated ATM phosphorylates Pex5, which promotes ubiquitination by Pex2 (E3 ligase) of Pex5 for the delivery of ubiquitinated peroxisomes to the autophagosome via p62 [197,198]. ATM also inhibits mTORC1 and promotes autophagy [94]. Moreover, peroxisomal H_2_O_2_ formation has been implicated in diseases such as diabetic nephropathy [199]. In LPS-induced AKI, impaired pexophagy and endothelial peroxisomal dysfunction promote oxidative damage in the kidney [200].

## 5. Concluding Remarks

Recent studies have begun to unfold the cross-talk between redox signaling and autophagy in renal diseases. Evidence suggests that multiple interacting pathways are involved between ROS and autophagy, and many cellular stresses increase ROS to regulate autophagy induction. The interplay between ROS and autophagy is highly relevant in renal diseases, particularly AKI and progressive kidney disease. The interaction between ROS and autophagy is regulated at both the transcriptional and translational level. In a posttranslational modification, ROS can modify many autophagy-related proteins by direct oxidation of cysteine residues, as redox regulation of Atg4 has been well documented. However, further studies are needed to identify this regulation of cysteine residue oxidation in renal diseases.

ROS and oxidative stress cause oxidative damage of cellular components and impair organelle function. Autophagy, in turn, eliminates dysfunctional organelles and damaged macromolecules and diminishes oxidative stress to maintain cellular homeostasis. However, under a disease state, dysregulated or impaired autophagy flux results in increased ROS production. Therefore, restoration of autophagic flux has remained a challenge when the level of oxidative stress increases during the progression of kidney diseases. More pharmacological approaches that can promote autophagic flux under disease conditions are required. Multiple studies have provided correlative evidence on the role of ROS in autophagy in renal diseases, however, further research must be performed to determine the underlying mechanisms and signaling pathways of the specific molecular interactions between ROS and autophagy. Additionally, the exact mechanisms behind the complex reciprocal relationship between oxidative stress and autophagy need to be elucidated in renal diseases. Autophagy plays an important role in both sensing oxidative stress and removing oxidatively damaged proteins and organelles, as well as the cellular machineries responsible for excessive ROS production. However, the precise mechanism describing how autophagy controls excessive ROS generation remains unknown.

Many studies on the impact of ROS on autophagy have been carried out in mTORC1-dependent induction of autophagy. The mTOR-independent autophagy-regulating pathways, including JNK-beclin-1-PI3KC3 pathways, as well as the p38, NFkB, Akt, Ca^2+^-calpain, and cAMP-Epac-PLC-ε-IP_3_ pathways, are relevant to kidney diseases. Future studies on the impact of ROS on these pathways will provide fruitful information. In addition, elucidation of the molecular mechanisms of interaction between ROS and autophagy, as well as identification of specific targets of ROS in the autophagy pathway, will provide a better understanding on the cross-talk between ROS and autophagy for the discovery of novel targets for renal disease prevention.

## Figures and Tables

**Table 1:** Effect of antioxidant/drug on autophagy in kidney disease.

Compound/Therapeutic Agent	Effect on Kidney Disease	Mode of Action	Reference
Rutin (Antioxidant flavonol glycoside)	Attenuated gentamicin-induced nephrotoxicity in rats	Reduced oxidative stress, autophagy, and inflammation	[61,62]
N-acetylcysteine (Antioxidant)	Ameliorated gentamicin-induced nephrotoxicity in miniature pigs	Reduced ROS and enhanced autophagy	[63]
Ferulic acid (antioxidant phenol)	Protected against experimental diabetic nephropathy in rats	Inhibited ROS, NF-κB, and stress signaling pathway (p38, JNK, ERK 1/2) activation, and promoted autophagy	[64]
Trehalose	Prevented cadmium-induced oxidative stress and subsequent cytotoxicity in the primary rat proximal tubule cells	Inhibited apoptosis and restored autophagic flux	[65]
Berberine (isoquinoline alkaloid)	Ameliorated cisplatin-induced renal dysfunction and histopathological changes in mice	Inhibited oxidative/nitrosative stress, inflammation, autophagy, and apoptosis	[66]
Aldosterone	Had a protective role in aldosterone-induced podocyte injury in mouse podocytes	Induced podocyte injury and simultaneously activated podocyte autophagy to protect against oxidative damage	[73]
Celastrol (antioxidant)	Protected against high glucose-induced injury, inflammation, and insulin resistance in podocytes in rats	Reduced inflammation and apoptosis and induced heme oxygenase (HO-1)-mediated autophagy	[67,68,69,70]

**Table 2:** Impact of antioxidants/drugs on AMPK-mTORC1-ULK1 pathway of autophagy in kidney disease.

Kidney Disease	Antioxidant/Drug	Effect on Kidney Disease	Mode of Action	Reference
Renal ischemia- reperfusion	Quercetin (flavonol antioxidant)	Protected against renal IR injury in mice	Increased AMPK-activation, inhibited mTOR activation, and activated autophagy	[126,127]
	Pioglitazone (Thiazolidinedione antioxidant)	Protected against renal IR in mice	Increased AMPK-regulated autophagy	[128,129]
	Omega 3-PUFA (Polyunsaturated Fatty Acids)	Protected against renal IR injury in mice	Increased AMPK-mediated autophagy	[130]
Cisplatin-induced nephrotoxicity	Ginsenoside Rb3	Protected against cisplatin-induced nephrotoxicity in mice	Reduced oxidative stress and autophagy via the AMPK/mTOR	[131]
	NAD(P)H: quinone oxidoreductase 1 (flavoprotein)	Protected against cisplatin-induced nephrotoxicity in vitro in NRK42-E cells	Enhanced autophagy via AMPK/mTOR signaling pathway	[132]
	Neferine (bisbenzylisoquinoline alkaloid)	Protected against cisplatin-induced nephrotoxicity in mice	Enhanced autophagy via the AMPK/mTOR signaling pathway	[133]
	Metformin (biguanide antihyperglycemic)	Ameliorated cisplatin-induced nephrotoxicity in mice	Enhanced autophagy via AMPK/mTOR signaling pathway	[134]
Podocyte damage	Ginsenoside Rb3	Protected mouse podocytes from aldosterone-induced injury by inhibiting ROS generation	Reduced aldosterone-induced autophagy via the AMPK/mTOR pathway in NRK-52E cells	[136]
	Bismuth nanoparticles (BiNP)	Increased ROS production in human embryonic kidney cells 293	Enhanced autophagy via the AMPK/mTOR signaling pathway	[135]
	Astragaloside IV (antioxidant)	Protection from podocyte injury in mice	AMPK-regulated autophagy induction	[141]
Cecal ligation and puncture-induced sepsis	SIRT3	Protected against AKI in a sepsis model of cecal ligation and puncture in mice	Enhanced autophagy via the AMPK/mTOR signaling pathway	[136]
Diabetic nephropathy	Berberine (isoquinoline alkaloid)	Reduced high glucose-induced apoptosis of mouse podocytes	Enhanced AMPK activation and autophagy	[137]
	Metformin	Protected against cisplatin-induced apoptosis in diabetic nephropathy in mice	Induced AMPK- regulated autophagy	[134]
	Mangiferin (polyphenol glucoside antioxidant)	Prevented diabetic nephropathy progression in mice	Protected podocyte function through autophagy via AMPK-mTOR-ULK1 pathway	[139]
	Cinacalcet (Type II agonist of calcium-sensing receptor)	Reduced oxidative stress and attenuated diabetic nephropathy in db/db mice	Modulated autophagy through the CaMKKβ-LKB1-AMPK pathway	[142]
